# Expanding the EVAR Pool with Non-IFU Patients: How Important is Subjective Physician Assessment?

**DOI:** 10.3390/jcm14041237

**Published:** 2025-02-13

**Authors:** Hasan Iner, Ismail Yurekli, Erturk Karaagac, Ihsan Peker, Nuri Utkan Tunca, Tahsin Murat Tellioglu, Huseyin Durmaz, Hidayet Onur Selcuk, Levent Yilik

**Affiliations:** 1 Department of Cardiovascular Surgery, Ataturk Training and Research Hospital, Izmir Katip Celebi University, 35360 Karabaglar, Izmir, Türkiye; hasan.iner@ikc.edu.tr (H.I.); ihsanpeker35@hotmail.com (I.P.); onur_selcuk_48@hotmail.com (H.O.S.); levent.yilik@ikc.edu.tr (L.Y.); 2 Department of Cardiovascular Surgery, Izmir City Hospital, 35510 Bayrakli, Izmir, Türkiye; ismoyurekli@yahoo.com; 3 Department of Cardiovascular Surgery, Pendik Training and Research Hospital, Marmara University, 34890 Pendik, Istanbul, Türkiye; utkan_tunca@yahoo.com; 4 Department of Cardiovascular Surgery, Hatay Training and Research Hospital, 31027 Antakya, Hatay, Türkiye; tmurattellioglu@gmail.com; 5 Department of Cardiovascular Surgery, Konya City Hospital, 42020 Karatay, Konya, Türkiye; durmazz1@hotmail.com

**Keywords:** abdominal aortic aneurysm, endoleak, EVAR, IFU, non-IFU, re-intervention

## Abstract

**Objectives:** In order to reduce the abdominal aortic aneurysm (AAA)-related complication rate in endovascular aneurysm repair (EVAR) procedures, manufacturers recommend following the instructions for use (IFU). However, IFU is considered too conservative in many centers. In this context, we present our experience and patient follow-up data with 248 consecutive patients with or without IFU eligibility. **Methods:** A total of 248 patients who underwent elective EVAR for AAA between 2014 and 2019 were included. In total, 190 patients were in the IFU group and 58 in the non-IFU group. Patients were evaluated for baseline demographic and anatomic data; unexpected periprocedural intervention; and postoperative data such as development of endoleaks during follow-up, need for re-intervention, development of complications, EVAR patency, and mean 5-year survival rate. **Results:** The patients did not differ in terms of basic demographic data. The basic anatomical data were suitable for the IFU standard. Intraoperative endoleak development was significantly higher in the non-IFU group. In addition, the development of endoleaks at any time, the need for re-intervention, and the development of complications were higher in the non-IFU group at postoperative follow-up. Survival analysis showed no difference in the mean 5-year follow-up. The EVAR patency rate was higher in the IFU group. **Conclusions:** We believe that the decision for a non-IFU EVAR should be patient-specific and that the results of the subjective medical assessment should definitely be taken into account. However, we should not forget that EVAR patients, especially non-IFU patients, are susceptible to future changes in the aorta and prone to the development of endoleaks and re-interventions.

## 1. Introduction

AAA is an aortic pathology that progresses rapidly, especially in association with comorbid factors, and has a mortality rate of up to 90% when rupture occurs [1]. The global prevalence of AAA in adults has been reported in studies to be 0.92% [2]. The treatment of AAA, which can have high mortality rates if left untreated, has improved significantly with EVAR, and EVAR treatment is increasingly preferred over open surgery (OSR) because it is less invasive [3,4].

To reduce the long-term AAA-related complication rate in EVAR procedures, it is recommended by the manufacturers to follow the instructions for use (IFU), which consist of anatomical rules that apply to the selected stent graft. However, the IFU is considered too conservative in many centers. On the other hand, it is often observed that EVAR is used in patients who are not listed in the IFU. In fact, studies have reported that almost one-fifth of patients in whom EVAR is used belong to the non-IFU group [5,6]. We can also say that in our current practice, the number of EVAR applications without endo-anchors in patients with hostile neck belonging to the non-IFU group is increasing day by day. However, the literature shows that early and late complication rates are higher in non-IFU cases [7,8].

In this context, we present our experience with 248 consecutive patients with or without IFU eligibility in our large-volume center. We report follow-up data aimed at finding differences in outcomes between the groups and other factors influencing mid- and long-term outcomes.

## 2. Patients and Methods

We performed EVAR using commercially available stent grafts in 402 consecutive patients with AAA between January 2014 and June 2019. A total of 57 patients who underwent emergency EVAR, 9 patients who underwent elective uni-iliac EVAR, 28 patients with missing follow-up data, 52 patients who underwent another commercially available stent graft, and 8 patients who underwent open conversion were excluded from the study. As a result, a total of 248 patients were included in the study. The study protocol was approved by the Katip Celebi University Faculty of Medicine Ethics Committee (2020-GOKAE-0610/IRB:1068). All patients gave written informed consent for anonymous collection of clinical data on the standard consent form provided by our institution. All data were obtained retrospectively from the hospital information registry system.

Inclusion criteria were patients who underwent elective bi-iliac EVAR due to AAA, which is an indication for surgical intervention according to the guidelines [9]. Exclusion criteria were a history of previous open abdominal aortic surgery, dissecting aortic aneurysm, isolated iliac artery aneurysm, emergency EVAR due to ruptured AAA, Fenestrated/Chimney EVAR, uni-iliac EVAR, or perioperative conversion to open surgery. In addition, patients whose postoperative follow-up data were not available were excluded from the study.

Before the procedure, all our patients underwent computed tomographic angiography (CTA) to evaluate aorto-iliac anatomy and plan the procedure. The procedure was planned by measuring the aneurysm diameter, neck diameter, neck length, angulations, and adequate distal and proximal landing zone length according to the previously approved method by the operators who performed the procedure after observing the 3-dimensionally reconstructed CTA images (3Mensio Vascular 4.2; Medical Imaging B.V., Maastricht, The Netherlands) [10,11].

Patients were assessed and recorded for compliance with the manufacturer’s IFU for the implanted device [12,13] (Table 1). The anatomical suitability of the proximal aortic necks for the stent graft was determined according to the IFU of the stent graft used [14]. If the proximal neck anatomical measurements were in accordance with the manufacturer’s recommendations for the endograft used, the patient group was included in the IFU group. Patients who were excluded from the IFU rules, including criteria such as aortic neck thrombus and neck anatomy, were included in the non-IFU group. For all procedures, two stent grafts with very similar IFU rules and operating mechanisms, Endurant II (Medtronic, Minneapolis, MN, USA) and Ankura (Lifetech Scientific, Shenzhen, China), were selected and used at the discretion of the operating physician.

All comorbidities of the patients were stable under medical treatment prior to elective EVAR surgery, and appropriate anesthetic techniques were used according to the patients’ comorbid factors.

All procedures were performed in the same hybrid operating room with surgical exploration of both common femoral arteries. All procedures were performed in accordance with the recommendations of the ESVS guidelines [9]. In all patients, the proximal landing site was planned to be as close as possible to the most caudal renal artery. Control aortography was performed before the procedure was completed. No patient left the operating room with a Type 1A or Type 3 endoleak.

Demographic characteristics, comorbidities, and anatomical features of the patients were defined and collected as preoperative data.

Intraoperative data were defined and collected as anesthesia method, aneurysm diameter, intraoperative unexpected interventions, additional interventions in the operating room, and operative time. Operative time was calculated considering the total time the patient stayed in the operating room. Intraoperative unexpected interventions, including all aortic and vascular access-related procedures (such as proximal cuff, distal extension, iliac PTA, iliac exploration for access, intervention for limb ischemia or vascular injury), were defined as intraoperative complications.

Postoperative data were defined and recorded as blood and blood product replacement, duration of mechanical ventilation, postoperative complications, ICU stay, hospital stay, presence of endoleaks and re-interventions, freedom from AAA complications, and length of follow-up period.

The patients in the IFU group underwent a CTA scan within the first 30 days postoperatively. CTA and/or a duplex ultrasonography (DUSG) scan was performed at the 6th month. Later, a DUSG scan was performed once a year during follow-ups, and CTA was performed when necessary. In the non-IFU group, DUSG was performed within the first 15 days due to the high risk of possible postoperative complications. BTA was performed in the 1st month, DUSG in the 3rd month, and CTA again in the 6th month. Then, annual BTA risks were taken into consideration and follow-ups were performed with DUSG and/or BTA protocol. Due to the risk of acute renal failure, follow-up CTA was not performed in patients with estimated glomerular filtration rates <45 mL/min, except for one month after the procedure. These patients were then evaluated with duplex ultrasonography to determine aneurysmal sac variations and endoleaks. Sac variation data were obtained from CTAs. All CTAs were evaluated by the same team that performed preoperative planning during patient follow-up.

All re-interventions and all postoperative complications related to AAA repair were reviewed. AAA-related complications were defined as all endoleak types, all causes of graft dysfunction, post-implant rupture, AAA-related death, thrombosis, limb ischemia, femoro-femoral graft complications, or the need for any other endovascular or surgical procedure. Primary endpoints were aneurysm-related mortality, procedure-related re-interventions, and the presence of Type 1 and Type 3 endoleaks.

## 3. Statistical Analysis

Statistical analysis was performed using IBM SPSS version 22.0 software (IBM Corp., Armonk, NY, USA). Continuous variables were expressed as mean ± standard deviation (sd), while categorical variables were expressed as number and frequency. The distribution of variables was measured using the Kolmogorov–Smirnov test. The Mann–Whitney U test was used to analyze quantitative independent data. The chi-square test was used to analyze qualitative independent data. A Kaplan–Meier survival analysis was performed to estimate the mean survival time of the sample. In addition, the log-rank test was performed to detect differences in survival distributions between groups. *p* < 0.05 was considered statistically significant.

## 4. Results

A total of 248 patients who underwent elective EVAR for AAA between January 2014 and June 2019 were included. A total of 190 patients were included in the IFU group and 58 in the non-IFU group. All patients had an infrarenal abdominal aortic aneurysm with an indication for intervention.

The mean age of the patients was 69.9 ± 8 years, and 93.9% were male. The most common concomitant disease was arterial hypertension, which was reported in 82.6% of patients. No statistically significant difference was found between the groups in terms of baseline demographic characteristics and comorbidities (Table 2).

The mean aneurysm size was 64.39 ± 12.8 mm, and there was no statistically significant difference in maximum aneurysm diameter between the groups (non-IFU: 67.1 ± 16.15 IFU: 63.57 ± 11.51). Proximal aortic neck characteristics were carefully evaluated and showed statistically significant differences in neck length, width, and angulation (*p* < 0.001). All values related to the proximal aortic neck, including calcium, thrombus, and overall neck morphology, were evaluated. Additional baseline anatomic characteristics are listed in Table 3.

When evaluating the periprocedural data, general anesthesia was the most frequently used anesthetic technique in both groups. Spinal and laryngeal mask anesthesia were statistically more frequently used in the IFU group. General anesthesia tended to be used in the non-IFU group.

Bilateral surgical access to both main femoral arteries was used in all patients. When the patient groups were analyzed for intraoperative complications, there was no statistically significant difference between the groups in terms of vascular injury and native limb occlusion. However, a significant difference was found in the non-IFU group compared to the IFU group in terms of intraoperative endoleaks. No difference was found with regard to other periprocedural data (Table 4).

No difference was found between the groups when evaluating the postoperative follow-up data in hospital. However, when evaluating the average 5-year follow-up data, the development of endoleaks was significantly higher in the non-IFU group. In addition, the evaluation of re-interventions after discharge showed that, statistically, more interventions were performed in the non-IFU group. The development of postoperative complications was significantly higher in the non-IFU group due to aneurysm-related complications. When EVAR patency was evaluated in relation to postoperative follow-up, EVAR patency was found to be significantly higher in the IFU group (Table 5). The Kaplan–Meier survival analysis, which was performed to assess the postoperative mortality of patients, showed no statistically significant difference. However, a trend towards mortality was observed in the non-IFU group towards the end of the 2nd year of follow-up (Table 6).

## 5. Discussion

The EVAR procedure was introduced a long time ago and has maintained its place in AAA treatment with successful applications. As a result, a significant amount of clinical data have been collected, including a large number of patients. Over the years, this clinical experience has led to the suspicion that IFU may be too conservative. It is well known that the anatomy of the aortic neck, which forms the basis of IFU, including the proximal landing zone length, diameter, angle, thrombus, and calcification rate, is the most important predictor of successful EVAR [15,16]. Thanks to developments in stent graft technology and product diversity over the years, new-generation grafts have made IFU even more liberal. This has made it possible to achieve effective and successful results even in unfavorable morphologies with standard endografts in non-IFU patient groups [5,6,17,18]. Our study found that 23.3% of patients enrolled in the study were treated with non-IFU EVAR without the use of endo-anchors.

The impact of patient demographic characteristics, particularly advanced age, female gender, and aneurysm sac size, on the long-term outcomes of EVAR patients has been demonstrated several times [19]. In our study, there was no statistically significant difference between the patient groups in terms of preoperative and demographic data. This allowed for the comparison of two homogeneous groups, which is the basis of the scientific work.

The first question that arises when using non-IFU EVAR is the success of the treatment. Once the graft is placed, the fact that the patency of the renal artery is maintained and there are no type I and III endoleaks is the basis for successful EVAR. In our study, no significant difference was found when the groups were compared in terms of intraoperative unexpected interventions. However, there was a statistically significant increase in intraoperative endoleaks in the non-IFU group (n: 5, 8.6%) compared to the IFU group (n: 4, 2.1%). In the non-IFU group, intraoperative endoleaks were detected in five patients (2 type IA, 3 type IB). Of these patients, one patient underwent aortic cuff placement, and two patients underwent iliac extension. In the other two patients, an aortic balloon was inserted. In the IFU group, four patients (one type IA, three type IB) had intraoperative endoleaks. These patients were treated with aortic balloons. No patient left the operating room with type 1 or type 3 endoleaks. Considering the anatomical characteristics of the non-IFU group, these results were not surprising. In the study by Beckerman et al. [20] involving 566 EVAR patients, 68.9% of whom were non-IFU patients, no statistically significant difference was found between the groups in terms of intraoperative insertion-related complications or other unexpected re-interventions. They reported that vascular complications occurred in about one-third of patients in both groups. The study by Elkouri et al. [21] reported that intraoperative re-interventions, including vascular access repair and embolectomy, were performed in 7.4% of patients.

In our study, there was a statistically significant difference between the study groups in terms of all endoleaks (IFU: 7.3% vs. non-IFU: 24.1%). In addition, re-interventions were statistically significantly higher in the non-IFU group. Although the endoleaks in the non-IFU group were repaired with endovascular procedures, endovascular intervention was not possible in the type 1A endoleaks detected in two patients in the IFU group, and these patients had to undergo aortobifemoral bypass surgery. When the patient groups were evaluated for AAA-related postoperative complications, this was found in 19 patients in the non-IFU group, and 17 of these patients were treated with endovascular re-intervention. In the IFU group, postoperative AAA-related complications occurred in 19 patients, and 17 of these patients were treated with endovascular re-intervention. Of the four patients who could not be treated with endovascular intervention in these two groups, two had massive gastrointestinal bleeding due to aortoenteric fistula, and the other two died due to AAA rupture. Endoleaks and re-interventions in the follow-up of EVAR patients have been the subject of many studies. Many studies in the literature report that early and late complications are higher in non-IFU EVAR cases, but there are also studies reporting the opposite [5,22,23]. The Endurant Stent Graft Natural Selection Global Post Market Registry (ENGAGE) study, which examined 1263 patients, reported that the IFU and non-IFU groups were comparable in terms of type 1 and type 3 endoleaks in the early postoperative period [24]. Hahl et al. [23] reported that, in their study of 258 EVAR patients, 114 (44.2%) of whom were non-IFU patients, there was no statistically significant difference in terms of all endoleaks and re-interventions. Accarino et al. [7] reported in their study comparing IFU and non-IFU EVAR patients that type 1A and secondary interventions were statistically significant in the non-IFU group.

The ARM rate is maybe the most the important criterion for the long-term patency of EVAR. In our study, there was no statistically significant difference between the groups in terms of ARM. The Kaplan–Meier survival analysis performed to compare the survival distributions between the groups showed no statistically significant difference. However, a trend was noted in terms of mortality in the non-IFU group, especially towards the end of the 2nd year. This demonstrates the fact that there were two groups with the same survival rates in the first 2 years. Therefore, it is justified to use the EVAR procedure in patients who do not fulfill the IFU criteria but are at a high risk for open surgical repair (OSR), even if they are non-IFU, instead of medical follow-up. In addition, this analysis predicts that non-IFU patients will have poorer long-term survival. Lee et al. [25] reported a statistically significant increase in the non-IFU group when comparing EVAR patients in terms of aneurysm-related mortality (ARM). A meta-analysis from 2020, which included 17 studies and 4498 patients, found a statistically significant increase in all-cause mortality in non-IFU patients [26]. Furthermore, De Martino et al. [27] reported in their study that they obtained similar results in EVAR patients who were not suitable for OSR. In our study, we believe that the small number of patients included in the study and the relatively short follow-up periods made it difficult for us to reach similar conclusions.

It is well known that reinterventions and intervention-free survival are reliable indicators of endograft patency. In our study, there was a statistically significant difference in long-term outcomes between the groups in terms of endoprosthesis patency (74.1% and 90.5%). However, in the study by Hwang et al. [28], intervention-free survival at 3 years was reported as 86% in patients treated with non-IFU, while it was reported as 96% in patients treated with IFU.

Like all our colleagues, we believe that the main goal in EVAR patency is to ensure endograft sealing in both IFU and non-IFU patients and to maintain this sealing even years after repair. Considering that the mean follow-up period of the patients included in our study was 5 years, we consider our results to be acceptable.

We accept that non-IFU patients have a higher risk of post-EVAR complications compared to IFU patients. However, due to the cost and difficulty of accessing branched or fenestrated EVAR procedures in countries like ours and the higher aneurysm non-related mortality risks in the non-IFU patient group, we believe that non-IFU EVAR intervention is the best option with acceptable risks instead of non-interventional follow-up. Our main motivation here is the aging of the patient group requiring intervention and the increase in comorbid factors. This explains our preference for EVAR as an alternative to major surgery such as OSR in the absence of alternative options despite the risk of re-intervention and long-term AAA-related complications. We also want to emphasize the importance of patient-centered subjective physician assessment, as stated in the study by George and colleagues [29]. We agree that the fact that IFU rules can be relaxed by physicians with sufficient intervention experience in experienced centers is a correct approach.

## 6. Limitations

There are some limitations to our study. First, it is a retrospective study. This led us to consider the current findings only as an association rather than a causal relationship. In addition, it is possible that some patients had follow-up and re-interventions outside our institution, for which we have no records.

Only two different commercially available stent graft brands were included in the study. This was because the IFU rules and application mechanisms were similar. This may have biased the study results.

In addition, the number of patients included in the study consisted of a relatively small sample size compared to larger registries. Prospective studies with larger patient numbers are needed on this subject.

## 7. Conclusions

It is known that the presence of comorbidities is a predictor of possible complications and mortality. There was no statistically significant difference in demographic data and mortality at 5-year follow-up between the groups included in our study. However, there was a statistically significant difference when endograft patency markers were evaluated. Nevertheless, we believe that non-IFU EVAR intervention is the best option with acceptable risks instead of non-interventional follow-up.

We think that individualization of preoperative and perioperative patient management and subjective medical assessment are necessary for better early and late postoperative outcomes in this patient group. However, further studies are needed to identify groups with lower risk of endoleaks and re-interventions to increase the safety of the procedure.

## Figures and Tables

**Table 1:** Instructions for use: anatomic criteria.

	Medtronic Endurant II/IIs	Lifetech Ankura
Proximal aortic neck diameter (mm)	19–32	18–32
Proximal aortic neck length (mm)	≥10	≥15
Proximal aortic neck angulation (degrees °)	≤60°	≤60°
Iliac artery length (mm)	≥15	≥15
Iliac artery diameter (mm)	8–25	8–20

**Table 2:** Baseline demographics and comorbidities.

	Overall n: 248	Non-IFU n: 58	IFU n: 190	*p* Value
	Mean ± s.d.	Mean ± s.d.	Mean ± s.d.	
Age	69.92 ± 8.06	71.06 ± 7.77	69.57 ± 8.14	0.230 ^M^
	n (%)	n (%)	n (%)	
Gender				0.347 ^C^
Male	233 (93.9%)	53 (91.3%)	180 (94.7%)	
Female	15 (6.1%)	5 (8.7%)	10 (5.3%)	
HLP	97 (39.1%)	21 (36.2%)	76 (40%)	0.604 ^C^
DM	41 (16.5%)	9 (15.5%)	32 (16.8%)	0.812 ^C^
HT	205 (82.6%)	46 (79.3%)	159 (83.6%)	0.441 ^C^
COPD	88 (35.4%)	23 (39.6%)	65 (34.2%)	0.448 ^C^
Smoking	131 (52.8%)	32 (55.1%)	99 (52.1%)	0.682 ^C^
PAD	49 (19.7%)	16 (27.5%)	33 (17.3%)	0.087 ^C^
CAD	96 (38.7%)	24 (41.3%)	72 (37.8%)	0.633 ^C^
CRF	26 (10.4%)	8 (13.7%)	18 (9.4%)	0.347 ^C^
CVE	24 (9.6%)	6 (10.3%)	18 (9.4%)	0.844 ^C^

HLP, hyperlipidemia; DM, diabetes mellitus; HT, hypertension; COPD, chronic obstructive pulmonary disease; PAD, peripheral artery disease; CAD, coronary artery disease; CRF, chronic renal failure; CVE, cerebrovascular event; *p* < 0.05; statistically significant; ^M^, Mann–Whitney U test; ^C^, chi-square test.

**Table 3:** Baseline anatomic features of aneurysm.

	Overall n: 248	Non-IFU n: 58	IFU n: 190	*p* Value
	Mean ± s.d.	Mean ± s.d.	Mean ± s.d.	
AAA diameter (mm)	64.39 ± 12.8	67.10 ± 16.15	63.57 ± 11.51	0.262 ^M^
Aortic bifurcation diameter (mm)	26.15 ± 2.38	27.6 ± 1.44	24.7 ± 2.26	<0.001 ^M^
Access femoral artery diameter (mm)	12.4 ± 2.43	12.1 ± 1.98	12.5 ± 2.05	0.545 ^M^
Lowest renal to aortic bifurcation length (mm)	113 ± 9.8	115 ± 13.4	112 ± 10.2	<0.421 ^M^
Neck length (mm)	16.84 ± 6.15	11.7 ± 3.22	19.98 ± 3.49	<0.001 ^M^
Neck diameter (mm)	25.4 ± 2.95	28.4 ± 2.48	25.4 ± 1.81	<0.001 ^M^
Infrarenal neck angulation (degrees °)	26.94 ± 10.53°	68.3 ± 11.02°	43.1 ± 13.38°	<0.001 ^M^
	n (%)	n (%)	n (%)	
Neck morphology				<0.001 ^M^
Straight	175 (70.5%)	20 (34.4%)	155 (81.5%)	
Conic	73 (29.5%)	38 (65.6%)	35 (18.5%)	
Neck calcium				0.934 ^C^
Absent	106 (42.7%)	23 (39.6%)	83 (43.6%)	
<90°	115 (46.3%)	28 (48.2%)	87 (45.7%)	
90–180°	24 (9.6%)	6 (10.3%)	18 (9.4%)	
>180°	3 (1.2%)	1 (1.7%)	2 (1%)	
Neck thrombus				0.663 ^C^
Absent	125 (50.4%)	29 (50%)	96 (50.5%)	
<90°	38 (15.3%)	11 (18.9%)	27 (14.2%)	
90–180°	54 (21.7%)	13 (22.4%)	41 (21.5%)	
>180°	31 (12.5%)	5 (8.6%)	26 (13.6%)	

AAA, abdominal aortic aneurysm; ^M^, Mann–Whitney U test; ^C^, chi-square test.

**Table 4:** Periprocedural data.

	Overall n: 248	non-IFU n: 58	IFU n: 190	*p* Value
	Mean ± s.d.	Mean ± s.d.	Mean ± s.d.	
Procedure durations (hours)	3.49 ± 1.01	3.62 ± 0.93	3.46 ± 1.03	0.522 ^M^
Number of stent graft components	2.7 ± 0.64	2.8 ± 0.60	2.6 ± 0.67	0.121 ^M^
Proximal oversize (%)	15.25 ± 2.9	15.3 ± 5.03	15.2 ± 2.7	0.610 ^M^
	n (%)	n (%)	n (%)	
Unexpected intervention need	27 (10.8%)	9 (15.5%)	18 (9.4%)	0.152 ^C^
Vascular injury	3 (1.2%)	1 (1.7%)	2 (1%)	0.682 ^C^
Native limb occlusion	14 (5.6%)	2 (3.4%)	12 (6.3%)	0.407 ^C^
Access need	1 (0.4%)	1 (1.7%)	0 (0%)	
Endoleaks	9 (3.6%)	5 (8.6%)	4 (2.1%)	0.020 ^C^
Anesthesia protocol				0.037 ^C^
General	143 (57.6%)	33 (56.8%)	110 (57.8%)	0.892 ^C^
Sedoanalgesia	67 (27%)	22 (37.9%)	45 (23.6%)	0.032 ^C^
Epidural	28 (11.2%)	2 (3.4%)	26 (13.6%)	0.031 ^C^
Laryngeal mask	10 (4%)	1 (1.7%)	9 (4.7%)	0.307 ^C^

^M^, Mann–Whitney U test; ^C^, chi-square test.

**Table 5:** Outcomes and postoperative data.

	Overall n: 248	Non-IFU n: 58	IFU n: 190	*p* Value
	Mean ± s.d.	Mean ± s.d.	Mean ± s.d.	
Length of Intubation (hours)	3.03 ± 3.02	3.17 ± 3.09	2.99 ± 3.01	0.610 ^M^
Blood product (unit)	1.31 ± 1.57	1.13 ± 1.33	1.37 ± 1.64	0.471 ^M^
Length of ICU stay (days)	1.50 ± 1.10	1.68 ± 1.40	1.44 ± 0.99	0.298 ^M^
Length of hospitalization (days)	5.07 ± 2.00	4.87 ± 1.56	5.13 ± 2.12	0.603 ^M^
	n (%)	n (%)	n (%)	
All endoleaks at follow-up	28 (11.2%)	14 (24.1%)	14 (7.3%)	<0.001 ^C^
Type 1A	10 (4%)	5 (8.6%)	5 (2.6%)	
Type 1B	12 (4.8%)	5 (8.6%)	7 (3.6%)	
Type 2	5 (2%)	3 (5.1%)	2 (1%)	
Type 3	1 (0.4%)	1 (1.7%)	0 (0%)	
All re-interventions at follow-up	34 (13.7%)	17 (29.3%)	17 (8.9%)	<0.001 ^C^
Embolectomy	5 (2%)	4 (6.8%)	1 (0.5%)	
Aortobifemoral bypass	2 (0.8%)	0 (0%)	2 (1%)	
Aortic cuff placement	8 (3.2%)	5 (8.6%)	3 (1.5%)	
Iliac limb extension	13 (5.2%)	6 (10.3%)	7 (3.6%)	
Peripheral revascularization	6 (2.4%)	2 (3.4%)	4 (2.1%)	
Postoperative complications	47 (18.9%)	20 (34.4%)	27 (14.2%)	<0.001 ^C^
AAA related *	38 (15.3%)	19 (32.7%)	19 (10%)	<0.001 ^C^
AAA non-related	9 (3.6%)	1 (1.7%)	8 (4.2%)	0.375 ^C^
Mortality in all times	43 (17.3%)	15 (25.8%)	28 (14.7%)	0.054 ^C^
AAA related	4 (1.6%)	2 (3.4%)	2 (1%)	0.204 ^C^
AAA non-related	39 (15.7%)	13 (22.4%)	26 (13.6%)	0.109 ^C^
Results				<0.001 ^C^
Patent EVAR	215 (86.6%)	43 (74.1%)	172 (90.5%)	
Non-Patent EVAR	33 (13.3%)	15 (25.8%)	18 (9.4%)	
Follow-up time (months)	63.53 ± 1.11	60.38 ± 2.49	64.49 ± 1.22	

*, includes patients with AAA related mortality; ^M^, Mann–Whitney U test; ^C^, chi-square test; ICU, intensive care unit; AAA, abdominal aortic aneurysm.

**Table 6:** Survival curves for EVAR procedures.

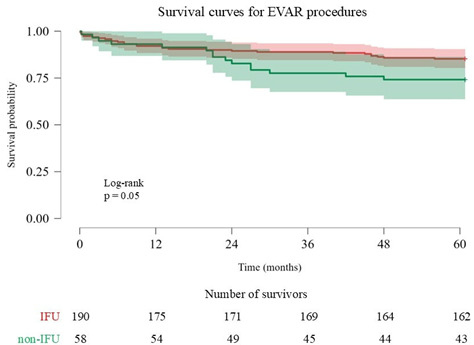					
Kaplan–Meier Survival Analysis					
Groups		Estimate	Std. Error	95% Confidence Interval	
				Lower Bound	Upper Bound
IFU	190	64.49	1.22	62.11	66.88
Non-IFU	58	60.38	2.49	55.49	65.27
Overall	248	63.53	1.11	61.36	65.70
Overall Comparisons					
			Chi-Square	df	Sig.
Log Rank (Mantel–Cox)			3.68	1.00	0.055
Breslow (Generalized Wilcoxon)			3.43	1.00	0.064
Tarone–Ware			3.56	1.00	0.059

## Data Availability

The data presented in this study are available on request from the corresponding author due to ethical reasons.
